# Effects of Decade Long Freezing Storage on Adipose Derived Stem Cells Functionality

**DOI:** 10.1038/s41598-018-26546-7

**Published:** 2018-05-25

**Authors:** Shahensha Shaik, Xiying Wu, Jeffrey Gimble, Ram Devireddy

**Affiliations:** 1 Bioengineering Laboratory, Department of Mechanical Engineering, Louisiana State University, Baton Rouge, LA USA; 2 La Cell LLC, Tulane University School of Medicine, New Orleans, LA USA; 3 Center for Stem Cell Research & Regenerative Medicine and Departments of Medicine, Structural & Cellular Biology, and Surgery, Tulane University School of Medicine, New Orleans, LA USA

## Abstract

Over the last decade and half, the optimization of cryopreservation for adipose tissue derived stromal/stem cells (ASCs) especially in determining the optimal combination of cryoprotectant type, cooling rate, and thawing rate have been extensively studied. In this study, we examined the functionality of ASCs that have been frozen-stored for more than 10 years denoted as long-term freezing, frozen within the last 3 to 7 years denoted as short-term freezing and compared their response with fresh ASCs. The mean post-thaw viability for long-term frozen group was 78% whereas for short-term frozen group 79% with no significant differences between the two groups. The flow cytometry evaluation of stromal surface markers, CD29, CD90, CD105, CD44, and CD73 indicated the expression (above 95%) in passages P1-P4 in all of the frozen-thawed ASC groups and fresh ASCs whereas the hematopoietic markers CD31, CD34, CD45, and CD146 were expressed extremely low (below 2%) within both the frozen-thawed and fresh cell groups. Quantitative real time polymerase chain reaction (qPCR) analysis revealed some differences between the osteogenic gene expression of long-term frozen group in comparison to fresh ASCs. Intriguingly, one group of cells from the short-term frozen group exhibited remarkably higher expression of osteogenic genes in comparison to fresh ASCs. The adipogenic differentiation potential remained virtually unchanged between all of the frozen-thawed groups and the fresh ASCs. Long-term cryopreservation of ASCs, in general, has a somewhat negative impact on the osteogenic potential of ASCs, especially as it relates to the decrease in osteopontin gene expression but not significantly so with respect to RUNX2 and osteonectin gene expressions. However, the adipogenic potential, post thaw viability, and immunophenotype characteristics remain relatively intact between all the groups.

## Introduction

Adipose tissue derived stromal/stem cells (ASCs), with an appropriate stimulus, can be differentiated into osteogenic, adipogenic, chondrogenic, myogenic, and neurogenic cell lineages^1–4^. Hence, ASCs have the potential to be used in cell based therapies to treat various diseases associated with bone^5–7^, heart^8–10^, kidney^11–13^, and neural tissues^14–16^. To store for future clinical use, ASCs are typically preserved using freezing techniques with the aid of cryoprotectants like dimethyl sulfoxide (DMSO), polyvinlypyrrolidone (PVP), methyl cellulose, etc^17–23^. Over the past few years, several studies have shown that the differentiation capacity, surface marker expression, proliferative capacity, and senescence of these cryopreserved ASCs remained virtually unchanged^20–26^. Most of these reported studies are done with the ASCs that are cryopreserved and stored for times ranging from 24 hours to up to one year. For clinical applications in the real world, the patient may require the ASCs after a decade or more from the point of donation^19,27,28^. However, the data on long term (at least ten years or more) effects of cryopreservation on ASCs has not as yet been reported in the literature and is the focus of the present study.

A study conducted on the peripheral blood progenitor cells stored for longer than 10 years reported the decrease in the viability and activity of red cell colonies and white cell colonies^29^. Likewise, prior studies have reported that the osteogenic potential of cryopreserved ASCs was found to be impeded both *in vitro* and *in vivo* in comparison to fresh ASCs^30^. Furthermore, it has been previously demonstrated that the age, BMI, and gender of the donor effect the ASC functionality^31–34^ and these factors might also impact the effects of long term cryopreservation storage outcomes. According to the International Federation for Adipose Therapeutics and Science (IFATS) and International Society for Cellular Therapy (ISCT), culture expanded ASCs must differentiate into adipogenic, chondrogenic, and osteogenic lineages and express surface markers CD73, CD90, CD44 and CD105^35^. Several other studies have reported that fresh ASCs express the surface markers CD73, CD 90, CD105, CD44 and CD29^36–38^. It is not known if the ASCs stored longer than ten years continue to meet the criteria set by the International Society for Cellular Therapy (ISCT) and retain the mesenchymal stem cell characteristics. Therefore, it is imperative to study the long term effects of cryopreservation on the ASCs to insure the development of safer and effective cell based therapies.

In this study, to determine the decade long effects of cryopreservation of ASCs, we investigated and compared ASCs processed from multiple donors, as shown in Table 1, that were cryopreserved for long-term (> = 10 years), short-term (3–7 years), and fresh ASCs (never cryopreserved). Specifically, we have investigated the post-thaw cell viability, stromal cell-surface markers, osteogenic and adipogenic differentiation potential of ASCs stored for periods ranging from 3 to 7 years (short-term) and 10 years or more (long term). The cell viability was assessed using live/dead staining, stromal cell surface markers with flow cytometry, osteogenic and adipogenic differentiation with histo-chemical staining and qPCR techniques.

**Table 1:** Classification of Donors Based the Freezing Storage Time*.

Group*	Donor	Year Frozen	Age	Sex	BMI
3–7 years	1	2012	47	F	34.8
	2	2009	57	F	21.3
	3	2014	34	F	33.0
> = 10 years	4	2005	32	F	27.4
	5	2006	47	F	29.9
	6	2007	52	F	23.7
Fresh	7	Fresh	27	F	24.4
	8	Fresh	51	F	31.2
	9	Fresh	52	F	23.8

*All the frozen-thawed experiments reported in this study were conducted in the year 2017.

## Materials and Methods

### ASC Cell Isolation and Culture

Protocols were reviewed and approved by the Pennington Biomedical Research Center Institutional Research Board or the Western Institutional Review Board. All methods were performed in accordance with the relevant guidelines and regulations. Lipoaspirate samples were obtained anonymously from de-identified patients undergoing elective liposuction procedures from the offices of plastic surgeons with written informed consent. The lipoaspirate samples from subcutaneous adipose tissue sites were processed within 24 hours of sample collection. The tissue was washed three to four times with pre-warmed phosphate-buffered saline (PBS) to remove erythrocytes and white blood cells. Floating adipose tissue fragments are separated from unwanted erythrocytes by removal of the infranatant solution. The tissue was then suspended in an equivalent volume of PBS containing 1% bovine serum albumin and 0.1% collagenase type I (Worthington Biochemical Corporation, Lakewood, NJ, www.worthington-biochem.com) that was pre-warmed to 37 °C. The solution was then placed in a 37 °C incubator with continuous agitation for one hour to enhance the digestion of the adipose tissue fragments. After digestion, the solution was centrifuged at 300 *g* for five minutes at room temperature to separate mature adipocytes from the stromal-vascular fraction (SVF). The solution was then homogenized by shaking and centrifuged again under the same conditions to enhance separation. The supernatant, containing lipids and primary, mature adipocytes, was then aspirated while the pellet was identified as the SVF containing adipose-derived adult stem cells. The SVF was suspended in stromal medium (Dulbecco’s modified Eagle’s medium [DMEM]/F-12 Ham’s (Hyclone Logan UT), 10%FBS (Hyclone, Logan UT, USA), 100 U penicillin/100 μg streptomycin/0.25 μg fungizone (Thermofisher scientific, CA, USA)) and centrifuged at 300 *g* for five minutes at room temperature to remove the remaining collagenase solution. The obtained pellet was suspended in stromal media and cultured in a cell culture incubator at 37 °C with 5% humidified CO_2_ as described elsewhere^39^.

### Cryopreservation/Freezing Protocol

The cultured ASCs were harvested from tissue culture flasks by using 0.25% trypsin-EDTA (Invitrogen, CA, USA) digestion, counted using a hemocytometer, and a million cells were suspended in the cryoprotectant medium containing 10% DMSO (V/V) in FBS solution. The cryovials were then placed in Mr Frosty^TM^ freezing container with iso-propanol which was then kept at −80 °C freezer overnight to achieve the cooling rate of 1 °C/min. The next day, the frozen vials were transferred to liquid nitrogen tank and stored starting from the year 2005 to 2014 (as shown in Table 1).

### Cell Viability

The frozen cryovials were thawed at 37 °C in a water bath by gently shaking for 1–2 minutes followed by diluting the cryoprotectant at 1:10 ratio by adding the stromal medium drop by drop (another 30 s). The samples were centrifuged at 300 g for 5 min to remove the cryoprotectant and the obtained pellet was suspended in 1 ml of stromal medium. The cells were stained with fluorescent dyes calcein AM at 2 µM and propidium iodide at 3 µM final concentrations. The number of live (green) and dead (red) cells were counted using hemocytometer under fluorescent microscope.

### Surface marker staining

ASCs at the respective passages were stained with antibody fluorophore conjugates CD146-PerCP-Cy5.5, CD29-PE, CD44-FITC, CD34-PE, CD105-FITC, CD90-FITC, CD31-Alexa Fluor 647, CD73-FITC, CD45-PE and their isotype controls. The antibody cocktails in the combination of CD146/CD29/CD44; CD34/CD105; CD90/CD31; and CD73/CD45 were prepared with final concentrations recommended by the manufacturer (All antibodies were purchased from BD Biosciences). Each antibody cocktail was added to suspension of 10^5^ cells and incubated for 20 minutes at 4 °C. Later, centrifugation was done to remove unbound antibodies and the cell pellet was suspended in 1% paraformaldehyde solution^40,41^ before flow cytometry (BD Biosciences, CA, USA) analysis on at least 10,000 cells per sample.

### RNA isolation, Reverse transcription, and qPCR

RNA was isolated using the purelink RNA kit (Life Technologies, CA, USA) according to manufacturer’s instructions. The quality and quantity of isolated RNA was measured using nanodrop spectrophotometer. The first strand cDNA synthesis was done by using high capacity cDNA synthesis kit (Applied biosciences, CA, USA). For quantitative real time PCR, a SYBR green (Applied Biosystems, CA, USA) kit was used as per the manufacturer’s instructions for the ABI-7900 qPCR machine. The primer sequences for osteogenic, adipogenic genes and cyclophilin B were described in Shah *et al*.^42^ and for GAPDH gene the primer sequence is (Forward 5′-GGTGTGAACCATGAGAAGTATGA-3′ and Reverse 5′-GAGTCCTTCCACGATACCAAAG-3′) which was designed by IDT primer quest tool. The fold change in gene expression was calculated and normalized to Cyclophilin B for adipogenic genes and to GAPDH for osteogenic genes by 2^−(Δ ΔCt)^ method^43^.

### ASC Differentiation

Frozen–thawed or fresh ASCs, cultured till passage 1 were plated in 12 well plates at a density of 3 × 10^4^ cells/cm^2^. Upon reaching 90% confluence, adipogenesis was induced by treating cells with adipogenic induction media, ADIPOQUAL™ (LaCell LLC, LA, USA) for ten days while replacing media for every three days. For control samples, stromal media containing DMEM with 10% FBS and 1% antibiotic was used. After 10 days, the adipogenic differentiated cultures were fixed in 4% paraformaldehyde and stained using the Oil Red O stain as described in several previous studies^17,19,21,22^. Quantitation of adipogenic staining was carried out by eluting the Oil Red O stain by 100% isopropanol followed by measuring absorbance of the elution at 510 nm. The amount of Oil Red O in the test samples was determined using a standard plot that was generated using known concentrations of Oil Red O.

Osteogenic differentiation, after reaching 90% confluence, was induced by treating ASCs with osteogenic medium, OSTEOQUAL™ (LaCell LLC, LA, USA) for 21 days by replacing media for every 3 days. For control samples, stromal media containing DMEM with 10% FBS and 1% antibiotic was used. To stain the deposited calcium phosphate in the extracellular matrix, the cells were fixed in 70% ice cold ethanol and stained with 2% alizarin red solution (pH adjusted to 4.1–4.3). Quantitation was performed by eluting the stain with 10% cetylpyridinium chloride followed by measuring absorbance of elution at 540 nm. The amount of alizarin red S in the test samples was determined using a standard plot that was generated using known concentrations of Alizarin Red S.

### Statistical Analysis

All the values are indicated as mean and standard deviation. Two tailed student’s t-test was employed, with P < 0.05 considered significant. The experiments were repeated three times on the ASCs derived from each donor. In total 9 different donors were evaluated with 3 donors per group (n = 3 per group).

### Data Availability Statement

The datasets generated during and/or analysed during the current study are available from the corresponding author on reasonable request.

## Results

### Post thaw viability

**Figure 1:**
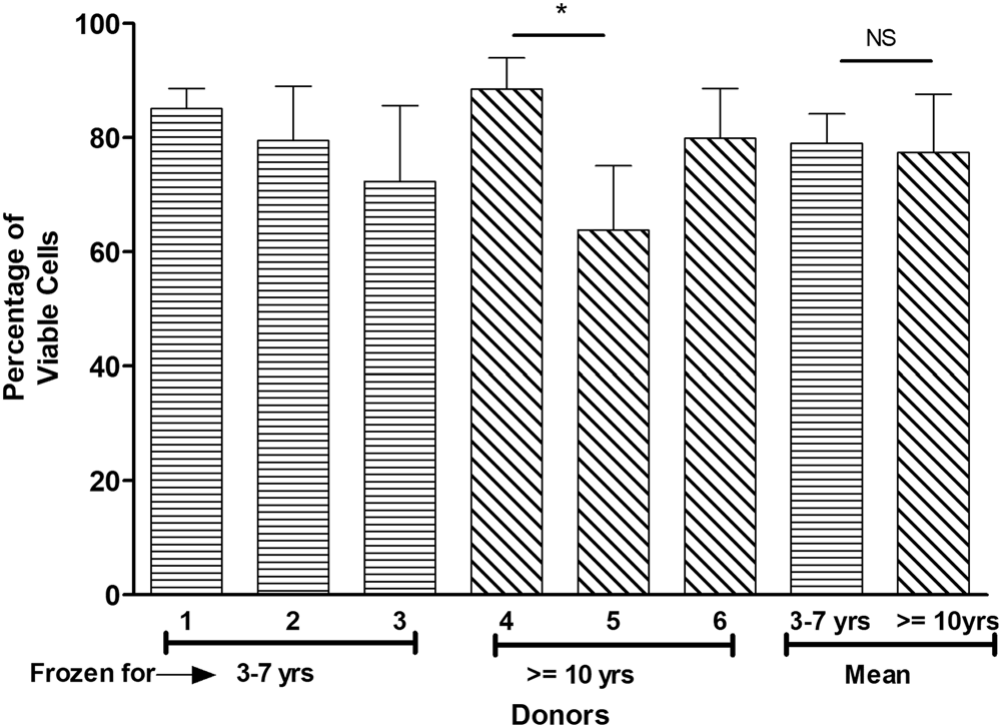
The post thaw viability of different ASC donors cryopreserved for short-term (3–7 years) and long-term (> = 10 years). The donor numbers (1–6) shown on the x-axis correspond to the numbers shown in Table 1. Additionally, the mean post thaw viability for each group (either the short-term or the long term is also shown in the last columns. The y-axis shows the percentage of viable cells obtained using live/dead assay with each donor measurement being made in triplicate (corresponding to the error bars shown). *Indicates P value < 0.05 as statistically significant. NS as not significant (P > 0.05).

The percentage of viable cells post thawing in both long-term and short-term groups was determined using calcein AM and propidium iodide fluorescent dyes. The mean cell viability for the donors in the short-term group was 79% whereas in the long-term group 78%. The cell viability percentages ranged from 63 to 88% across the donors and no statistical significance was observed indicating that the cryopreservation storage time has no effect on cell viability (Fig. 1).

### Surface marker characterization

**Figure 2:**
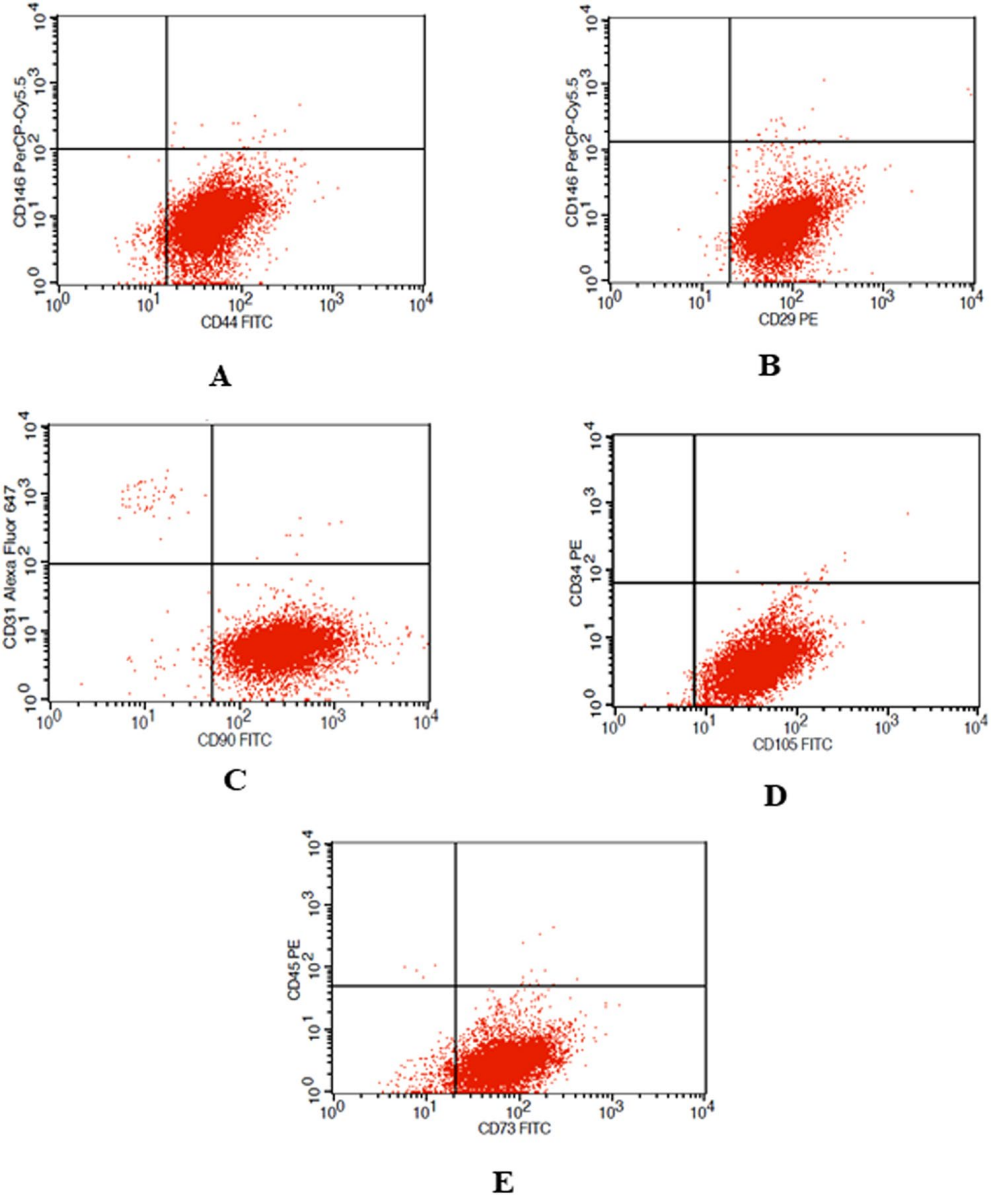
The flow cytometry dot plots for stromal cell (CD29, CD90, CD105, CD44, and CD73) and hematopoietic markers (CD31, CD34, CD45, and CD146) from a representative donor at Passage 1 are shown. (**A**) shows the CD44 (x-axis) and CD146 (y-axis); (**B**) shows CD29 (x-axis) and CD146 (y-axis); (**C**) shows CD90 (x-axis) and CD31 (y-axis); (**D**) shows CD105 (x-axis) and CD34 (y-axis); € shows CD73 (x-axis) and CD45 (y-axis).

**Figure 3:**
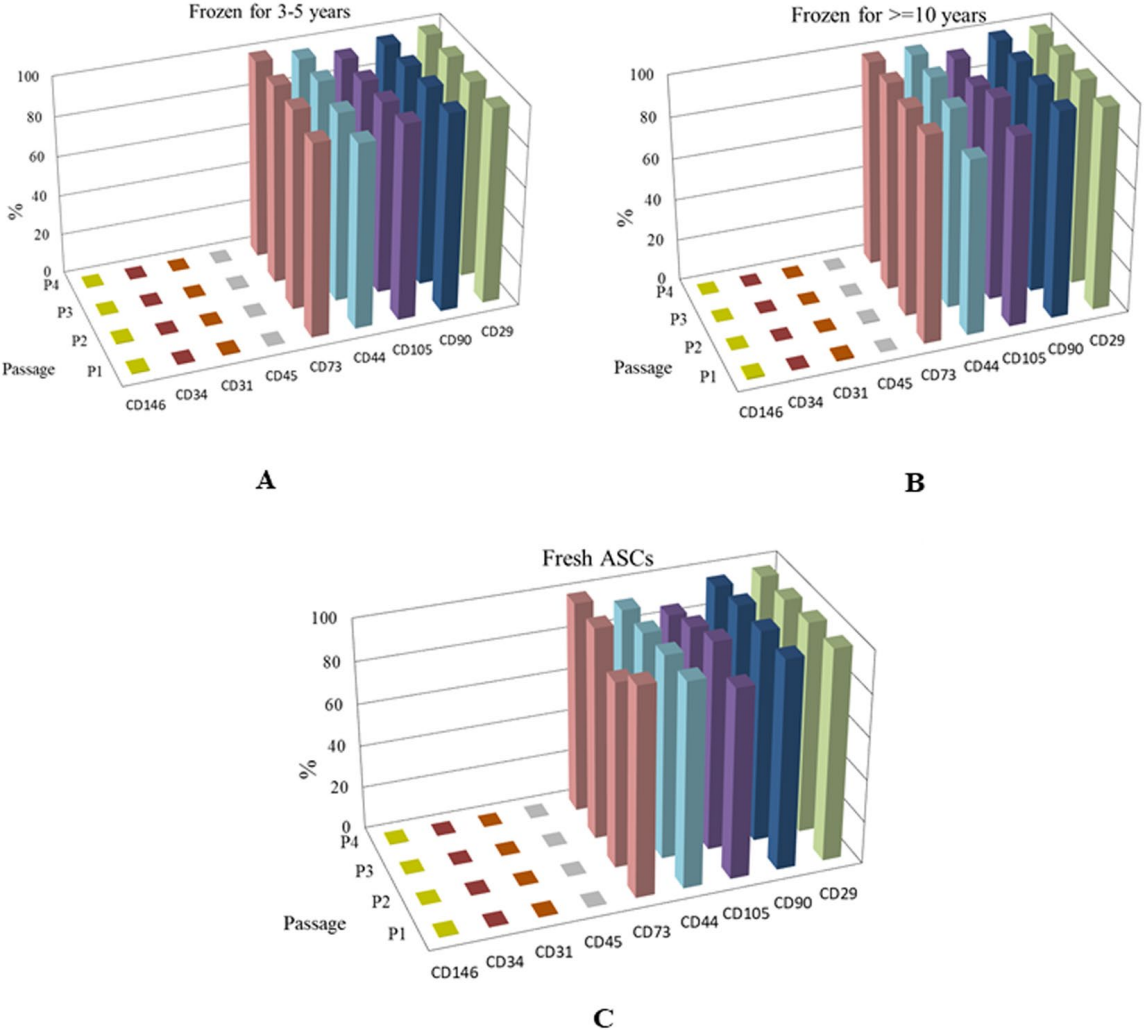
The mean percentage of surface marker expression on ASCs determined by flow cytometry for the passages P1-P4 are shown. (**A**) short-term (3–7 years) frozen-thawed cells; (**B**) long-term (> = 10 years) frozen-thawed cells; (**C)** Fresh ASCs. The figures depict passage number on the z-axis, the surface marker expression percentage on the y-axis while the x-axis shows the stromal (CD29, CD90, CD105, CD44, and CD73) and hematopoietic (CD31, CD34, CD45, and CD146) surface markers. No significant differences were found between the groups and between the various donors.

The expression of surface markers was evaluated by flow cytometry at the respective passages from P1-P4. The representative flow cytometric images are presented in (Fig. 2). The stromal cell-markers CD29, CD90, CD105, CD44, and CD73 expressed at above 90% consistently through the passages P1-P4. Although there are few instances where the expression of CD44 at P1 from the long-term group and the CD105 at P1 and P4 from fresh group were found lower than 90%, the statistical significance was not reached (Fig. 3, Table 2). The expression levels of the endothelial cell surface markers CD31 and CD146, hematopoietic markers, the common leukocyte antigen CD45, and CD34, were virtually undetectable. The trend of higher expression of stromal cell-markers and unnoticeable levels endothelial and hematopoietic markers is observed through the passages 1–4 across all the donors, and between different groups regardless of the cryopreservation and freezing storage. Moreover, no significant differences were found in the surface marker expression in short-term cryopreserved, long-term cryopreserved, and fresh ASCs groups suggesting that the immunophenotype of the ASCs is virtually unaffected by the cryopreservation and the time length the cells spend in ultralow temperatures.

**Table 2:** Surface Marker Expression Through Passages 1–4 of Fresh and Cryopreserved ASCs Stored for Various Time Periods.

Surface Markers	3–7 years				10 years				Fresh			
	P1 (n = 3)	P2 (n = 3)	P3 (n = 3)	P4 (n = 3)	P1 (n = 3)	P2 (n = 3)	P3 (n = 3)	P4 (n = 3)	P1 (n = 3)	P2 (n = 3)	P3 (n = 3)	P4 (n = 3)
CD146	0.52 ± 0.38	0.61 ± 0.51	0.26 ± 0.24	0.29 ± 0.23	0.87 ± 1.27	0.25 ± 0.19	0.19 ± 0.07	0.16 ± 0.07	0.32 ± 0.11	0.03 ± 0.05	0.15 ± 0.14	0.38 ± 0.30
CD29	97.58 ± 2.54	98.42 ± 1.27	99.24 ± 0.63	99.10 ± 0.50	97.29 ± 3.41	98.88 ± 1.39	99.72 ± 0.24	98.98 ± 0.77	99.55 ± 0.19	99.34 ± 0.67	98.26 ± 1.10	97.73 ± 2.09
CD44	92.23 ± 2.30	94.49 ± 2.85	97.92 ± 1.06	97.33 ± 3.35	84.05 ± 18.96	95.80 ± 3.55	99.01 ± 0.27	98.82 ± 1.13	96.26 ± 3.48	95.65 ± 1.66	93.75 ± 2.74	92.64 ± 5.90
CD34	0.17 ± 0.14	0.16 ± 0.11	0.260.14	0.196 ± 0.09	0.25 ± 0.21	0.13 ± 0.08	0.12 ± 0.07	0.12 ± 0.12	0.19 ± 0.05	0.17 ± 0.17	0.06 ± 0.05	0.19 ± 0.15
CD105	97.66 ± 1.58	95.90 ± 2.51	94.44 ± 8.49	93.86 ± 7.52	91.06 ± 4.79	97.00 ± 3.49	91.64 ± 8.47	93.60 ± 7.98	89.42 ± 17.60	98.03 ± 1.69	92.61 ± 1.32	86.61 ± 13.36
CD90	98.95 ± 0.41	99.62 ± 0.09	98.2 ± 1.49	97.37 ± 3.30	99.03 ± 1.34	99.64 ± 0.17	99.76 ± 0.11	99.27 ± 0.93	98.55 ± 1.04	99.05 ± 1.19	99.12 ± 1.00	96.66 ± 4.51
CD31	0.38 ± 0.32	0.14 ± 0.10	0.05 ± 0.03	0.03 ± 0.03	0.93 ± 1.31	0.21 ± 0.16	0.12 ± 0.07	0.08 ± 0.06	0.40 ± 0.27	0.09 ± 0.06	0.05 ± 0.03	0.04 ± 0.02
CD73	96.24 ± 4.64	99.77 ± 0.06	99.47 ± 0.27	99.75 ± 0.18	98.82 ± 1.32	99.26 ± 1.05	99.88 ± 0.11	98.83 ± 0.80	98.19 ± 1.73	87.17 ± 17.54	99.53 ± 0.36	99.28 ± 0.29
CD45	0.27 ± 0.24	0.19 ± 0.12	0.553 ± 0.42	0.283 ± 0.19	0.05 ± 0.03	0.72 ± 0.99	0.23 ± 0.22	0.07 ± 0.02	0.32 ± 0.14	0.08 ± 0.04	0.16 ± 0.17	0.34 ± 0.53

### Osteogenic potential

**Figure 4:**
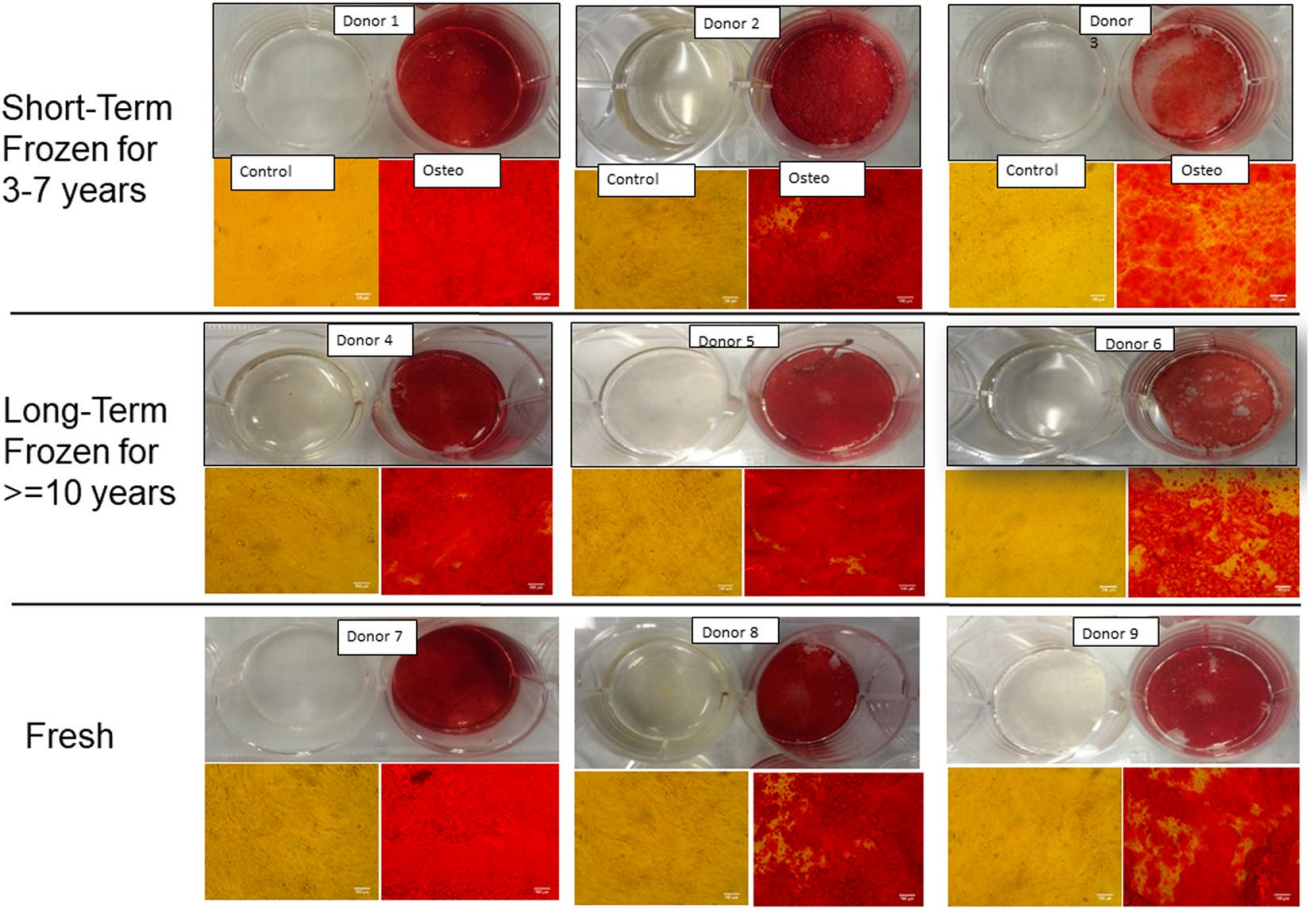
Comparison of osteogenic differentiation by Alizarin Red S between various donors. Top Row: Short-term (3–7 years) frozen-thawed ASCS; Middle Row: Long-term (> = 10 years) frozen-thawed ASCs; Bottom Row: Fresh ASCs. The donor numbers (1–9) shown on the figures correspond to the numbers shown in Table 1. Alizarin Red S staining was performed after 21 days of osteogenic induction on osteogenic differentiated samples and their controls. The images are of magnification 10x and the scale bar represents 100 µm.

**Figure 5:**
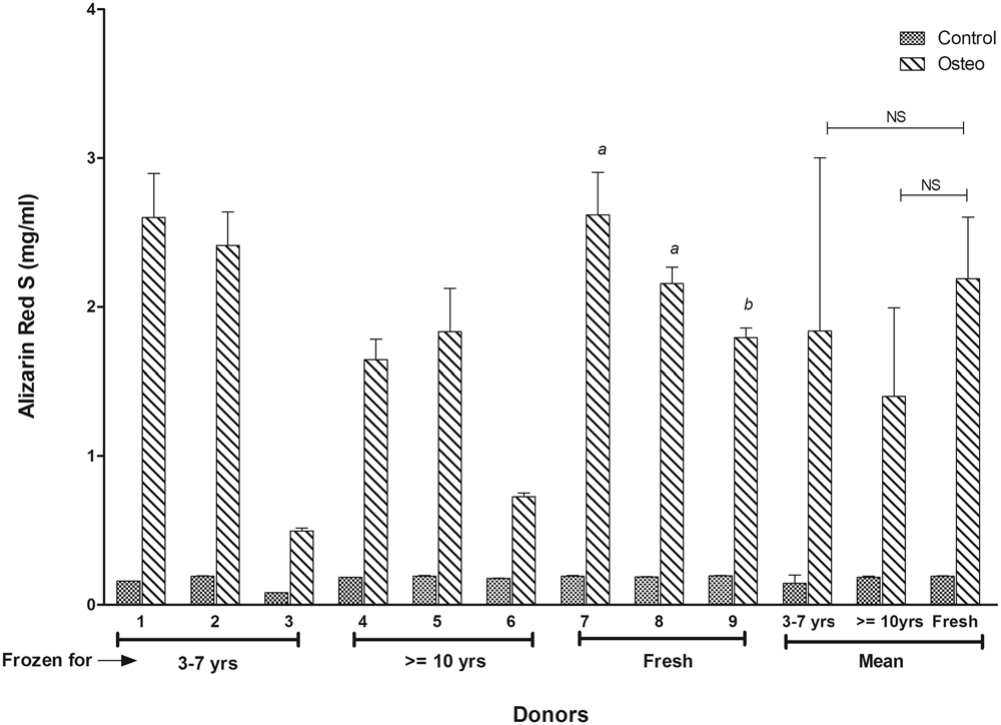
Quantitation and comparison of Alizarin Red S staining from the osteogenic differentiated short-term (3–7 years) frozen-thawed ASCs, long-term (> = 10 years) frozen-thawed ASCs, and fresh ASCs. The donor numbers (1–9) shown on the x-axis correspond to the numbers shown in Table 1. The y-axis shows the concentration of alizarin red stain (mg/ml) as described in the text with each donor measurement being made in triplicate (corresponding to the error bars shown). “a” indicates P value < 0.05 relative to donors 3, 4, and 6. “b” indicates P value < 0.05 relative to donors 3 and 6. “NS” indicates no statistical significance between the means of Fresh group in relation to 3–7 years and > = 10 years group.

**Figure 6:**
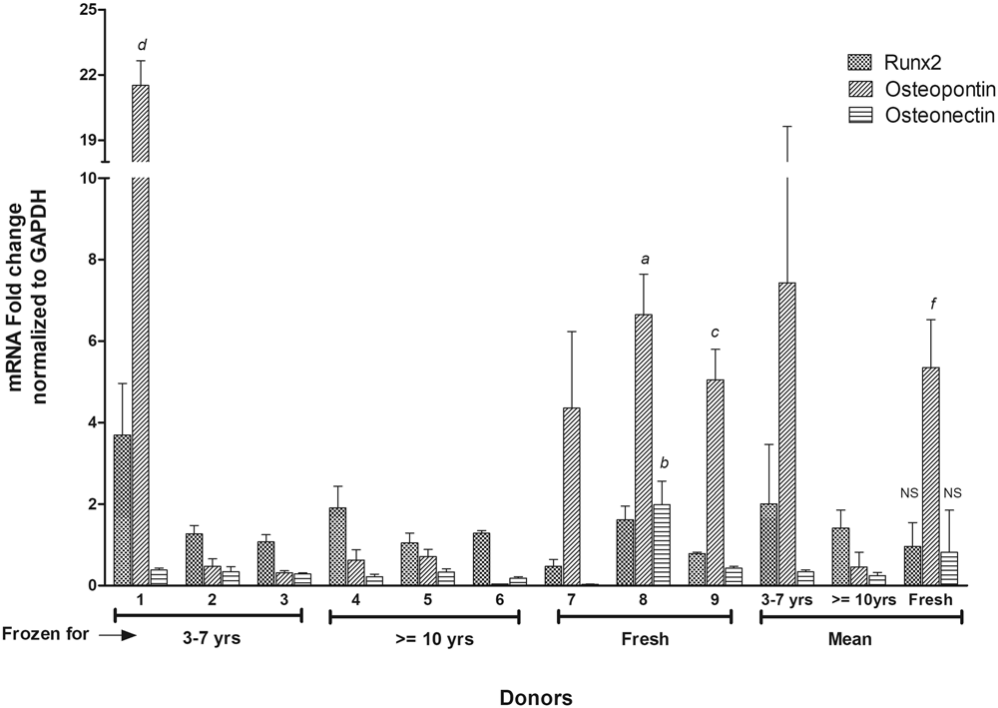
qPCR Osteogenesis— the expression of osteogenic genes RUNX2, Osteonectin, and Osteopontin for short-term (3–7 years) frozen-thawed ASCs, long-term (> = 10 years) frozen-thawed ASCs, and fresh ASCs. The donor numbers (1–9) shown on the x-axis correspond to the numbers shown in Table 1. The y-axis shows the mRNA fold change normalized to GAPDH as described in the text with each donor measurement being made in triplicate (corresponding to the error bars shown). The decrease in the osteogenic gene expression in relation to the fresh ASCs was found in long-term and short-term frozen groups. Notice that the ASCs from donor #1 in the short-term frozen-thawed group shows a higher expression than fresh ASCs. “a” indicates P value < 0.01 relative to donors 2, 3, 4, 5, and 6. “b” indicates P value < 0.05 relative to donors 2, 3, 4, 5, and 6. “c” indicates P value < 0.02 relative to donors 2, 3, 4, 5, and 6. “d” indicates P value < 0.005 relative to donors 7, 8, and 9. “f” indicates P value < 0.02 relative to the mean of > = 10 yrs group for the gene Osteopontin. “NS” indicates no statistical significance between the means of fresh group in relation to 3–7 yrs and > = 10 yrs groups for the genes RUNX2 and Osteonectin.

ASCs treated with osteogenic medium for 21 days were evaluated by histochemical staining with Alizarin Red S and qPCR to examine the expression of osteogenic genes. The photomicrographs for the histochemical staining of osteogenesis for all the donors are presented in (Fig. 4). The Alizarin Red S stain quantitation indicates that the amount of staining found in majority of the donors in long-term and short-term cryopreserved groups is comparable to the amount of staining observed with fresh ASC group (Fig. 5). However, one donor from each group (donor 3 and donor 6), displayed relatively lower staining relative to other donors (Fig. 5). No statistical significance was found with the Alizarin Red S stain quantitation between the donor means of the groups. The osteogenic gene expression especially that of osteopontin, was found to be increased in all the donors from the fresh group (Fig. 6). RUNX2 and osteonectin were both upregulated in donor 8. Most of the donors from the long-term and short-term cryopreserved groups showed minimal increase in RUNX2 whereas the expression of osteopontin and osteonectin remained at basal levels with no increase (Fig. 6). However, donor 1 from the short-term group, displayed a substantial increase in the expression of RUNX2 and osteopontin surpassing the expression levels of all other donors in the fresh group (Fig. 6). Statistical analysis of the donor mean values between the groups revealed strong statistical significance (P < 0.02) in the increase of osteopontin gene expression in the fresh group with respect to long-term group. However, no statistical significance was found with RUNX2 and osteonectin expression between these two groups. In addition, there were no significant differences between fresh and short-term groups. These results suggest a weak but probable correlation between the loss of osteogenic gene expression and time/length of freezing storage.

### Adipogenic potential

**Figure 7:**
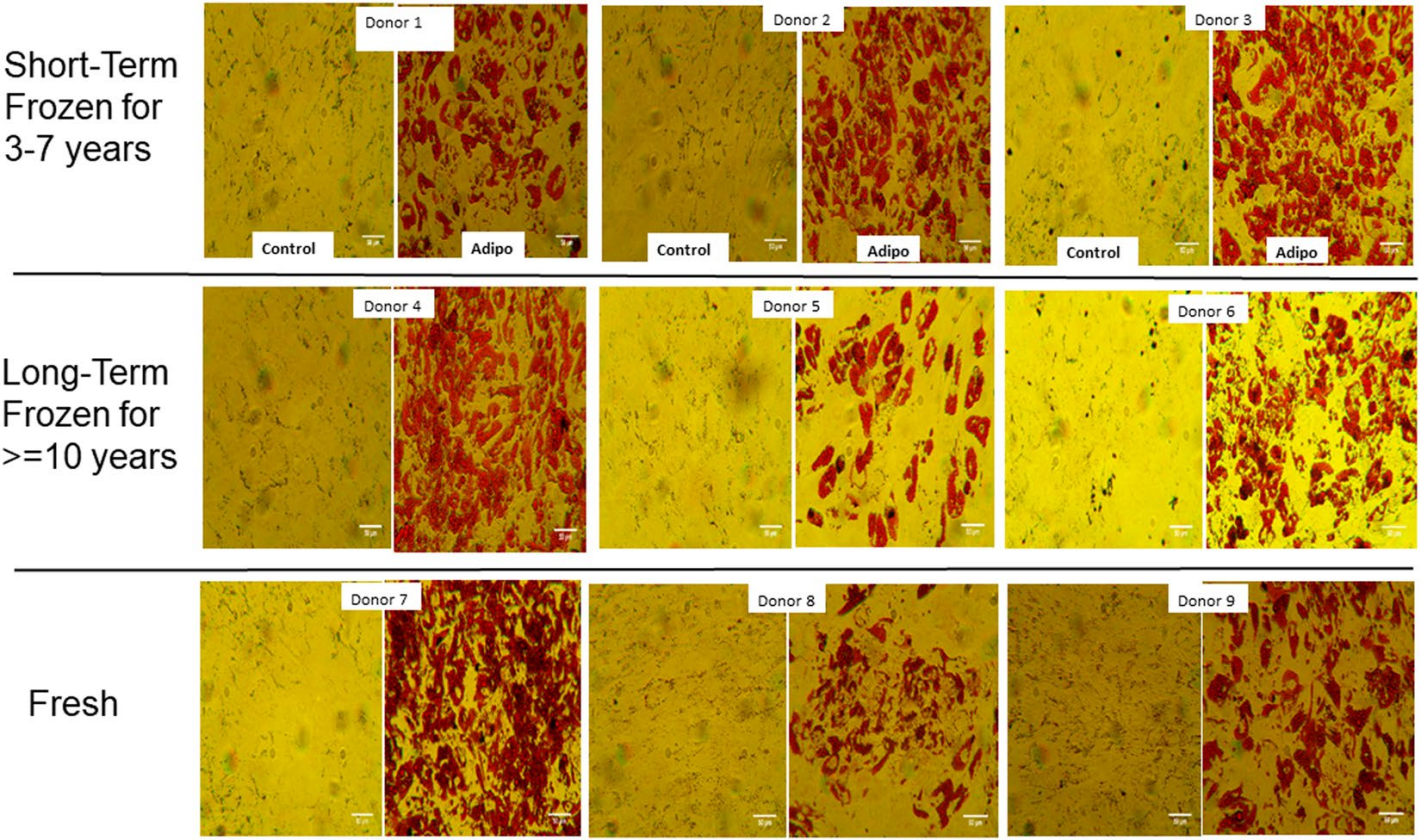
Comparison of adipogenic differentiation by Oil Red O staining between various donors. Top Row: short-term (3–7 years) frozen-thawed ASCs; Middle Row: long-term (> = 10 years) frozen-thawed ASCs; Bottom Row: Fresh ASCs. The donor numbers (1–9) shown on the figures correspond to the numbers shown in Table 1. Oil Red O staining was performed after 10 days of adipogenic induction on adipogenic differentiated samples and their controls. The images are of magnification 25x and the scale bar represents 50 µm.

**Figure 8:**
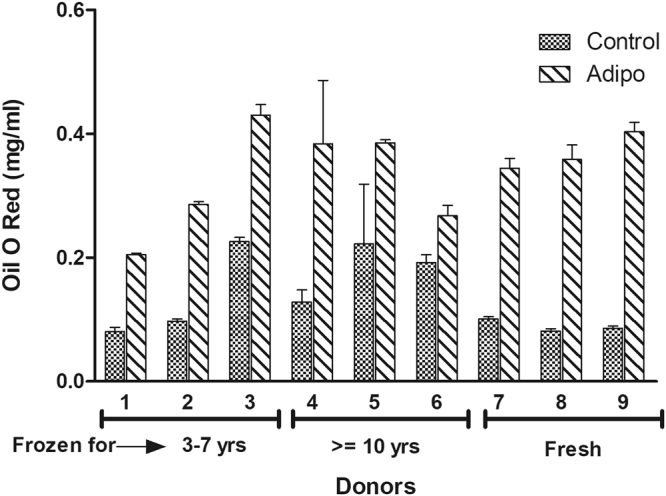
Quantitation and comparison of Oil Red O staining from the adipogenic differentiated short-term (3–7 years) frozen-thawed ASCs, long-term (> = 10 years) frozen-thawed ASCs, and fresh ASCs. The donor numbers (1–9) shown on the x-axis correspond to the numbers shown in Table 1. The y-axis shows the concentration of Oil Red O staining (mg/ml) as described in the text with each donor measurement being made in triplicate (corresponding to the error bars shown). No significant differences in gene expression between long-term and short-term frozen groups in relation to the fresh ASCs is observed.

**Figure 9:**
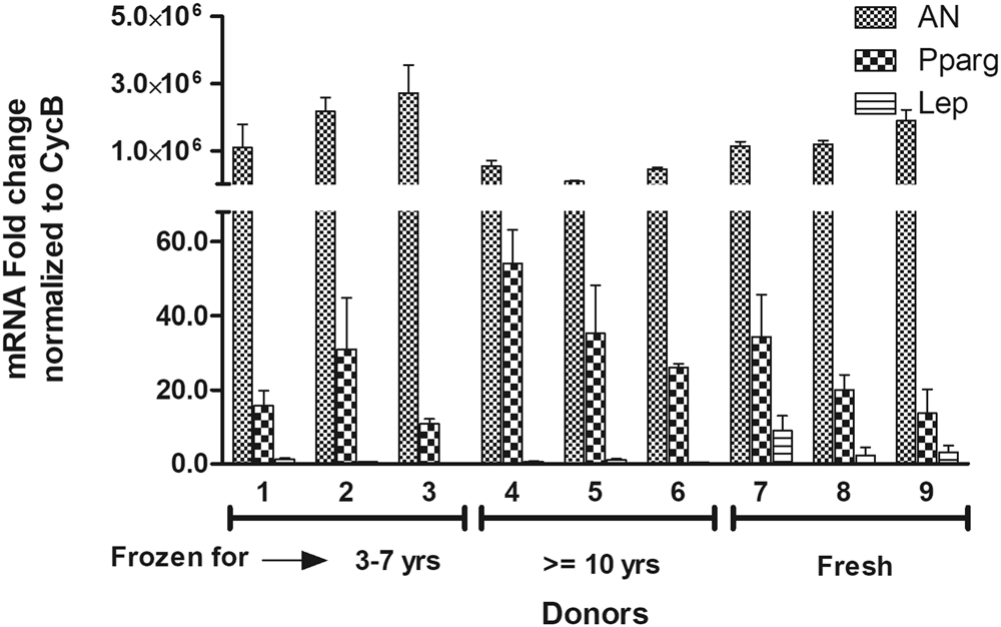
qPCR Adipogenesis— the expression of adipogenic genes Adiponectin (AN), PPARg, and Leptin (Lep) for short-term (3–7 years) frozen-thawed ASCs, long-term (> = 10 years) frozen-thawed ASCs, and fresh ASCs. The donor numbers (1–9) shown on the x-axis correspond to the numbers shown in Table 1. The y-axis shows the mRNA fold change normalized to CycB as described in the text with each donor measurement being made in triplicate (corresponding to the error bars shown). No significant differences in gene expression between long-term and short-term frozen groups in relation to the fresh ASCs is observed.

ASCs treated with adipogenic medium for 10 days were evaluated by histochemical staining with Oil Red O and qPCR to examine the expression of adipogenic genes. The photomicrographs for the histochemical staining of adipogenesis for all the donors are presented in (Fig. 7). The Oil Red O stain quantitation indicates that the amount of staining in found in majority of the donors in long-term and short-term cryopreserved groups is comparable to the amount of staining observed with fresh ASC group with no significant differences observed (Fig. 8). The expression of adipogenic genes especially adiponectin (AN) and PPARg were found to be increased in all the donors regardless of cryopreservation and freezing storage (Fig. 9). Leptin expression was found to be minimally induced in the majority of donors (Fig. 9). Overall, this data suggests that adipogenic potential of ASCs remains unchanged with or without the cryopreservation and the storage period in liquid nitrogen.

## Discusssion

Based on the theoretical analysis it has long been hypothesized that cells stored at −196 °C would remain virtually intact at least for millennia due to the cessation of all bio-chemical reactions except for the meager ionizing effects from the background radiation^44–46^. This hypothesis was proven correct in case of sperm cells where several studies have reported that the human semen cryopreserved for several decades, in one reported study up to 40 years, have led to successful live births following artificial insemination^47–49^. To this extent, this theory seemed to be applicable for every cell type, however, long-term cryopreservation studies conducted on bone marrow cells and peripheral blood progenitor cells (PBPCs) delineated conflicting picture on the functionality of the long-term frozen cells.

Overall, despite conflicting results, there is some consensus in published literature on the effects of long-term cryopreservation of bone marrow cells or PBPCs and suggests a loss of functionality with increase in freezing storage time as well as the storage conditions; specifically, storage in dry ice or higher storage temperatures than in liquid nitrogen do lead to cell degradation and loss of functionality. For example, PBPCs frozen and stored in the vapor phase of liquid nitrogen for 15 years resulted in suboptimal activity and reduced viability of white and red blood cells whereas no significant differences were found in samples stored up to 10 years^29^. Human bone marrow stored for overs ten years in the vapor phase of liquid nitrogen have also been shown to form lower colony forming units in culture (CFU-C) and burst forming unit-erythroid (BFU-E) capacity although higher viable cells were found post thawing^50^. Conversely, a few other studies on bone marrow cells frozen and stored up to 14 years in liquid phase of liquid nitrogen reported retention of CFU-C capability with minimal loss of viability^51,52^. A comparative study done on bone marrow frozen for 2 or 5 months the vapor phase of liquid nitrogen found that the minimal dosage of canine bone marrow required for effective autologous engraftment was higher with cells frozen and stored for 5 months suggesting a decreased recovery and lower cell viability than cells frozen and stored for 2 months frozen as well as fresh bone marrow cells^53^. It is also important to note that freezing storage conditions have a significant impact on freezing storage outcomes. For example, human bone marrow cells frozen in glycerol and stored in dry ice for more than 3 years shown increased damaged compared to the cells stored in liquid nitrogen^54^. Although, it should still be noted that this study by Malinin *et al*.^54^, also showed that cells stored in liquid nitrogen were also sub-optimal when compared to fresh cells. Conversely, frozen storage of cord blood cells in liquid nitrogen for 15 years showed the retention of CFU-C potential with no negative effects on proliferative and cytotoxic responses^55^. Similarly, short term cryopreservation of bone marrow cells up to 3 years at −90 °C resulted in no significant difference of cell viability, but did exhibit a modest decrease in CFU-C forming potential^56,57^.

The critical factors that influence the outcome of stem cell freezing are the type of cryoprotectant, cooling rate, and warming rate^58–64^. Several studies have been performed to optimize these parameters on ASCs^18,20,65^. However, most of these studies were solely focused on optimizing one of these above mentioned parameters and the effect of long-term freezing storage time was largely left unexplored. For example, exposure of human embryonic stem cells to DMSO was found to cause key changes in stem cell markers, protein content, and functionality^66^. Similarly, ASCs cryopreserved with DMSO and PVP and stored for 2 weeks at −196 °C maintained their osteogenic and adipogenic potentials as well as their post thaw viability^21,39^. We have recently reported that thermally pre-conditioned ASCs cryopreserved in either DMSO or PVP and stored for 1 day at −196 °C displayed enhanced cell viability while retaining their differentiation plasticity^22^. Cryopreservation of human ASCs for 3 months in liquid nitrogen displayed no sign of deterioration in their functionality regarding immunophenotype, differentiation potential, proliferation, and viability^67^. Investigation of 4-year long cryopreserved ASCs in liquid nitrogen revealed the homologous expression of stromal cell-surface markers from passages P2-P8 with the potential to differentiate into mesenchymal tri-lineage^68^. Porcine ASCs stored for at least 1 year in liquid nitrogen with 10% DMSO have not expressed any negative changes related to osteogenic and adipogenic potential, immunophenotype, and growth characteristics^25^. Similarly, canine ASCs frozen and stored for 1 year in liquid nitrogen showed no changes in the osteogenic, adipogenic, and chondrogenic potentials^69^.

To our knowledge this is the first study to investigate the cryopreserved ASCs stored over a decade. We found that the ASCs from passages 1 to 4 express high percentage of stromal cell-markers especially CD29, CD105, CD44, CD90, and CD73 in the long-term frozen group. The comparison between long-term, short-term frozen with the fresh ASCs revealed no significant differences regarding the immunophenotype expression. Mitchell *et al*.^36^ reported that the SVF from the adipose tissue is comprised of heterogeneous cell population with various cell subsets expressing hematopoietic markers CD31, CD34, and CD146 along with stromal cell-markers. With the increase in the passage number, ASCs acquire homogeneity which results in the loss of expression of hematopoietic markers with a corresponding increase of stromal cell markers^36^. We performed the immunophenotype evaluations from passage 1 and ASCs in all the groups displayed homogeneity with higher stromal marker expression and minimal expression of hematopoietic markers, as shown in Table 2 and Fig. 3. This observation is in agreement with Mitchell *et al*.^36^. Conversely, we found extremely low percentages hematopoietic markers at P1 than were reported in Mitchell *et al*.^36^. This lack of hematopoietic/endothelial markers CD31, CD146, and CD34 can partially be attributed to the static nature of cell culture with minimal hydro- or hemo-dynamic forces^70^. Baer *et al*.^71^ detected the expression of CD34+ ASCs between passages P2-P4 in some donors but not in all. Consequently, Baer *et al*. concluded that CD34 expression is extremely variable between donors and correlates to their medical history and cell culture conditions^71^. Since no discernible differences in the immunophenotype expression were observed between long-term frozen, short-term frozen, and fresh ASCs it can be concluded that decade long cryopreservation virtually has no impact on the surface marker expression of ASCs.

The differentiation potential evaluated by histochemical staining and gene expression quantitation indicated that the measured difference in the adipogenic capabilities of long-term/short-term frozen ASCs in comparison with the fresh ASCs are not statistically significant. However, the osteogenic potential of cryopreserved ASCs was impaired in the long-term cryopreserved group as was evident with loss of osteopontin gene expression in comparison to fresh group. Interestingly, no significant differences were observed in the expression of RUNX2 and osteonectin between the mean values for donors between the various frozen groups. We should be careful to note that RUNX2 is an early osteogenic marker^72^ and since we performed qPCR after 21 days of osteogenic differentiation it is quite possible that RUNX2 expression retreated to basal level at the point of evaluation. Additional experiments are required to assess the expression of RUNX2 at earlier time points during the osteogenic differentiation pathway. In addition, other notable osteogenic genes such as osteocalcin, Bone Morphogenetic Protein 2 (BMP2) and osterix weren’t evaluated in our study. Studying the expression of these genes could further corroborate or reject our weak observation in the loss of osteogenic potential associated with freezing and the length of low temperature storage time. A similar decrease in the osteogenic potential of frozen-thawed ASCs has also been reported previously by James *et al*.^30^. Further studies are clearly needed to evaluate whether the reduced osteogenic potential of the long-term cryopreserved ASCs can be enhanced if the post-thaw media is supplemented with additional growth factors or by thermal pre-conditioning of cell culture^22^.

Additionally, the current study indicates that expression of stromal cell-markers does not necessarily correlate with the differentiation potential of cryopreserved ASCs. Our data indicates that ASCs irrespective of the freezing-storage time exhibit > 95% stromal marker expression typical of fresh ASCs and yet have disproportionately reduced osteogenic potential. This observation is consistent with findings by Lo Surdo and Bauer^73^ and Lo Surdo *et al.*^74^ that quantitative analysis of surface antigens alone may be insufficient to accurately assess the differentiation capacity and functionality of mesenchymal stem cell populations. Overall, these findings underscore the importance of reconsidering the use of long-term frozen-thawed ASCs for osteogenic clinical applications and highlight the potential challenges involved with their clinical translation.

## Conclusion

In this study, we evaluated and compared the functionality of ASCs cryopreserved for long-term (> = 10 years) and short-term (3–7 years) to fresh ASCs. In total, 9 different donors were studied with 3 donors in each group. Post-thaw viability, immunophenotype and differentiation potential were examined. We found that the osteogenic differentiation potential was partially hampered in the long-term cryopreserved group as exhibited by osteopontin gene expression as well as in the short-term freezing group (with the exception of cells from one anomalous donor that showed robust osteogenic potential) in comparison to fresh ASCs. The osteonectin and RUNX2 gene expression were not significantly different between the various groups. Additionally, the adipogenic potential of the frozen-thawed cells remained unaffected by freezing as well as the length of the storage time. Immunophenotype and post-thaw viability of ASCs also remained intact after decade long freezing process in relation with fresh ASCs. Finally, this study shows that the expression of stromal-cell markers should not be conclusively considered as a definitive benchmark indicator of the plasticity of cryopreserved ASCs.
